# Ozonation of ketoprofen with nitrate in aquatic environments: kinetics, pathways, and toxicity

**DOI:** 10.1039/c7ra12894k

**Published:** 2018-03-16

**Authors:** Yongqin Zeng, Xiaoxuan Lin, Fuhua Li, Ping Chen, Qingqing Kong, Guoguang Liu, Wenying Lv

**Affiliations:** School of Environmental Science and Engineering, Guangdong University of Technology No. 100 Waihuan Xi Road, Guangzhou Higher Education Mega Center, Panyu District Guangzhou 510006 China liugg615@163.com; School of Environment and Chemical Engineering, Foshan University Guangdong 528000 China; School of Environment, Tsinghua University Beijing 100084 China; School of Environmental Science and Engineering, Sun Yat-Sen University Guangzhou 510275 China

## Abstract

In this study, nitrate ion (NO_3_^−^) was found to collaborate with ozone thereby accelerating the degradation of ketoprofen. NO_3_^−^ was discovered to induce the generation of hydroxyl radicals (·OH), which was crucial to the decomposition of PPCPs in wastewater treatment plants. Kinetic studies on the decomposition of ketoprofen were investigated under different concentrations of NO_3_^−^. The impact mechanisms and degradation by-products were experimentally determined. The results revealed that all reactions fitted the pseudo-first-order kinetic model well. The presence of NO_3_^−^ had the capacity to accelerate the ozonation of ketoprofen. The reaction by-products were evaluated by UPLC-Q-TOF-MS, and a total of five intermediates generated *via* the ozonation of ketoprofen were assessed. The transformation pathways were concluded to be hydroxylation, nitration, and debenzophenone and ketonized reactions. Additionally, the toxicity of the by-products was evaluated by employing *Chlorella* and *Daphnia magna*.

## Abstract 2

In this study, nitrate ion (NO_3_^−^) was found to collaborate with ozone thereby accelerating the degradation of ketoprofen.

## Graphical abstract

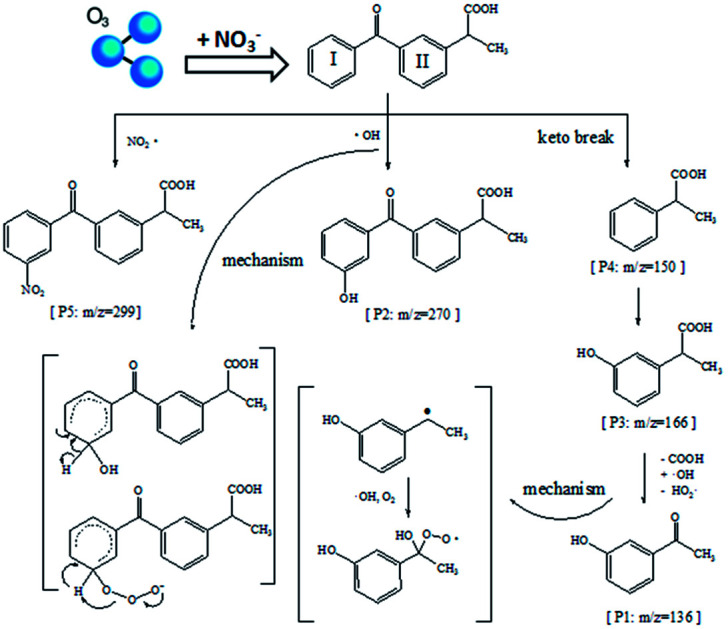

## 1 Introduction

Recently, pharmaceuticals and personal-care products (PPCPs) have provoked much public consideration as “emerging contaminants”.^1^ Such ubiquitous pollutants may cause potential risks to human health due to their biologically active natures, accumulation, and persistent physicochemical properties.^2^ Among the PPCPs, ketoprofen ([2-(3-benzoylphenyl) propionic acid]; KET) is one of the most widely used non-steroidal anti-inflammatory drugs (NSAIDs), which is commonly employed in clinics to treat rheumatoid arthritis, non-rheumatic diseases, ankylosis spondylitis as well as osteoarthritis or postoperative pain.^3^ Due its relatively high production, widespread usage, direct or indirect emissions, and low removal efficiency, ketoprofen has been investigated to a significant degree in wastewater treatment plants, surface waters, groundwaters, and even drinking water sources at home and abroad. Santos^4^ pointed out that the mean concentration of ketoprofen approached 2.50 μg L^−1^ in wastewater effluents in Spain. Furthermore, PPCPs found in the River Taff and River Ely in the UK were investigated, among which ketoprofen was found at concentrations that ranged between 0.5 and 14 ng L^−1^. Moreover, Cao found that the maximum concentration of ketoprofen in the Zhangweinanyun River system was 31.35 ng L^−1^,^5,6^ whereas concentrations in the Yuecheng Reservoir ranged from 1.33 to 8.40 ng L^−1^. Additionally, the expanding aging “Baby boomer” population and improving quality of life worldwide means that overall consumption of ketoprofen is likely to increase over the next few years.^7^ Nevertheless, such adducts are difficult to effectively remove *via* conventional drinking water treatment processes including filtration, sedimentation and flocculation. Subsequently, certain contaminants may still be present during the disinfection step of the drinking water treatment process.

Ozonation is a promising and environmentally compatible oxidation process. It is favorable for use in drinking water disinfection for its ability to degrade various recalcitrant organic pollutants through the direct use of ozone (O_3_, *E* = 2.07 V) and indirect use of hydroxyl radicals (·OH, *E* = 2.8 V).^8–10^ Several studies have revealed that ozone shows great capacity in decomposing PPCPs.^11–13^ To the best of our knowledge, information regarding ketoprofen being efficiently eliminated by the ozonation process in pure water has been long known for a long time.^14,15^ However, until now, detailed research pertaining to the degradation of target pollutants under the repercussions of coexisting ions has been negligible.

Inorganic nitrogen (including primarily nitrate, nitrite, and ammonium ions) has been frequently detected in both surface and groundwaters at concentrations of 10^−6^ to 10^−3^ M.^16,17^ Over the last few years, inorganic nitrogen has been extensively studied, and it has been proven that these nitrogen-containing ions may produce hydroxyl radicals and other reactive groups, thereby promoting or restraining the oxidation of dissolved organic pollutants.^18,19^ As a matter of fact, NO_3_^−^ has been considered as an environmental impact factor instead of a major research object in most studies.^20,21^ Thus, it is critically important to investigate the impact of NO_3_^−^ on ketoprofen degradation by ozone during the drinking water disinfection process.

Given the lack of data and knowledge in this field, the goals of this study were to (1) technically evaluate the influence of NO_3_^−^ toward the removal of ketoprofen by ozone under simulated water disinfection conditions including an elucidation of both the reaction kinetics and overall mechanism; (2) identify the disinfection by-products of KET with NO_3_^−^ present in the system, presuming a tentative degradation pathway for KET during the process; (3) simply evaluate the toxicity of the by-products with the employment of *Chlorella* and *D. magna*.

## 2 Materials and methods

### 2.1 Reagents

Ketoprofen, (KET, 2-(3-benzoylphenyl) propionic acid (purity ≥ 98%)) and ibuprofen were both purchased from TCI Reagent Co. Ltd. (Shanghai, China). Acetonitrile of HPLC-grade was purchased from U. S. ACS Enke Chemistry Co. Ltd. (Guangzhou, China). Benzoic acid was obtained from Aladdin Industrial Co. Ltd. (Shanghai, China). Other reagents employed in this study (*e.g.*, sodium nitrate, sodium nitrite, ammonium sulfate, sulfuric acid, sodium hydroxide, and acetic acid) were all of analytical grade and were used without further purification. These reagents were obtained from the Guangzhou Chemical Reagent Factory. Ultrapure water (18.25 MΩ cm^−1^), obtained from a Milli-Q apparatus (Smart2 Pure ultrapure water/water system integration, TKA, Germany), was used in the preparation of all aqueous solutions during the experiment.

### 2.2 Experimental apparatus and procedures

**Fig. 1:**
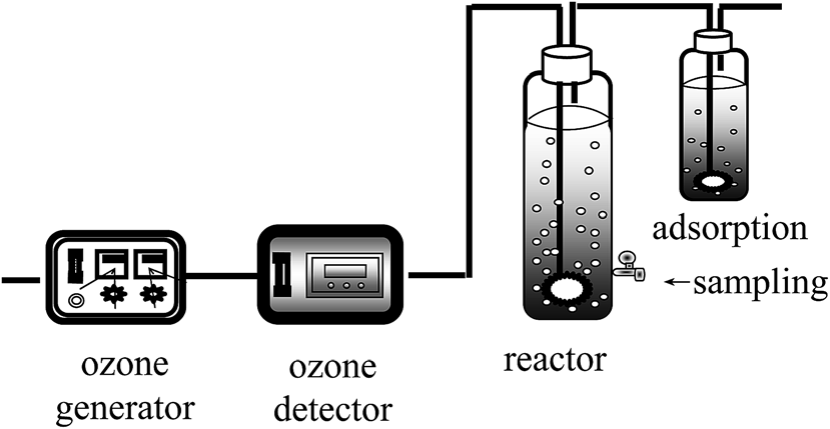
Reaction equipment.

500 mL of 20 μmol L^−1^ KET solution was prepared, and the initial pH values were adjusted by NaOH or H_2_SO_4_ solution to keep the initial pH of each sample consistent and to reduce the experimental error. As shown in Fig. 1, the reactions were initiated by the introduction of ozone gas from an ozone generator (Quanju Technology, China), after which the gas was passed into an ozone concentration detector (UT-500, Adel Measurement and Control Technology Co., LTD, USA) where the ozone content in mixed gas was determined to be 16 μmol L^−1^. Degradation experiments were conducted with laboratory-fabricated glass equipment (Fig. 1) using a gas dispersing device with a tap at the bottom. Ultimately, the unused portion of the gas mixture was absorbed by the potassium iodide solution.

### 2.3 Analytical methods

During the course of the experiments, 1.5 mL volumes of reaction liquid were incrementally extracted from the tap at 2, 4, 8, 12, 16, and 24 min. The samples were immediately analyzed *via* the reversed-phase high-performance liquid chromatography system (Waters, USA), which was equipped with a Zorbax Eclipse XDB-C18 column (4.6 × 150 mm, 5 μm, Agilent, USA). The analytical column temperature was 40 °C, and 45% HPLC-grade acetonitrile and 55% Milli-Q-Water (containing 0.5% glacial acetic acid) were used as the mobile phase at a constant flow rate of 1.0 mL min^−1^. The injection volume was 10 μL, and the UV wavelength for detection was 260 nm. The elution time of KET was approximately 9.0 min.

The KET degradation by-products were determined with the UPLC-Q-TOF-MS microsystem (Waters, USA) under chromatographic conditions (Table 1). The chromatographic column was introduced into a UV-vis detector equipped with an electrospray ionization (ESI) interface and mass analyzer, and the experiment was performed using a Waters TQ detector. The electrospray source voltages were as follows: capillary (3.0 kV), sample cone (30 V), and extraction cone (4.0 V) under negative mode. The source block and desolvation gas were heated at 100 and 300 °C, respectively. The concentrations of NO_3_^−^ were determined by ion chromatography (882, Metrohm AG, Switzerland).

**Table 1:** Chromatographic conditions

Time (min)	Flow (mL min^−1^)	*A*%	*B*%
0	0.3	95	5
6	0.3	80	20
13	0.3	0	100
14	0.3	95	5
16	0.3	95	5

## 3 Results and discussion

### 3.1 Ozonation performance: ketoprofen influenced by NO_3_^−^

**Fig. 2:**
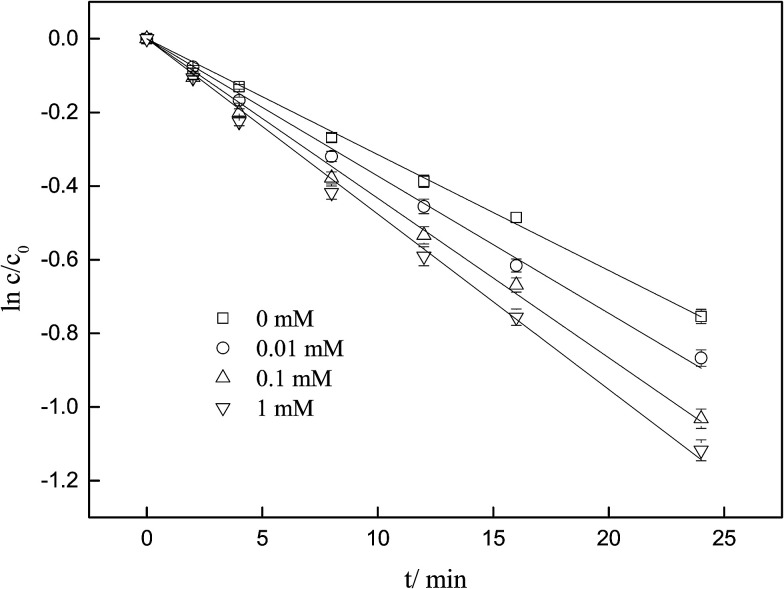
Influence of NO_3_^−^ on ozonation of KET.

As displayed in Fig. 2, the degradation of ketoprofen fitted pseudo first-order kinetics well. The degradation rate constants were 3.0 × 10^−2^, 3.6 × 10^−2^, 4.2 × 10^−2^ and 4.6 × 10^−2^ min^−1^ when the concentrations of NO_3_^−^ were 0, 0.01, 0.1, and 1 mmol L^−1^, respectively; the corresponding percentages (*η*) were calculated to be 53%, 58%, 64%, and 70%. The results indicated that ketoprofen reacted effectively with O_3_, and more than half of the ketoprofen used for this study was degraded within 24 min. It could be inferred that further degradation might be achieved with the prolongation of the reaction time. It was observed that NO_3_^−^ could accelerate the ozonation of ketoprofen, and as the concentration of NO_3_^−^ was increased, a stronger effect was elicited. The same phenomenon occurred when Miao investigated the effect of NO_3_^−^ on the ozonation of phenazone.^22^

Typically, the degradation of organics by ozone is initiated through the combined activities of ozone molecules and hydroxyl radicals that are decomposed from O_3_;^23^ the mechanism is illustrated in eqn (1) and (2). As previous studies have indicated, NO_3_^−^ might induce the generation of ·OH to promote the degradation of organic matter in an oxidation system;^18^ the mechanism is articulated in eqn (3).

1 O_3_ + OH^−^ → HO_2_^−^ + O_2_

2 O_3_ + HO_2_^−^ → ·OH + O_2_˙^−^ + O_2_

3 NO_3_^−^ + O_2_˙^−^ + 2H_2_O →·NO_3_ + 2·OH + 2OH^−^

To further explore the mechanism of influence of NO_3_^−^ on the ozonation of ketoprofen, quenching experiments were conducted to identify the generation of ·OH. *tert*-Butyl alcohol (TBA), a widely used ·OH scavenger in many quenching experiments,^10,24,25^ was selected to confirm the existence of ·OH in the system and to evaluate the contribution of ·OH to the degradation of ketoprofen in accordance with eqn (4). As exhibited in Table 2, TBA dramatically reduced the degradation of ketoprofen, which indicated that ·OH existed in the system and contributed to the degradation of ketoprofen, with the contribution rates of 90%, 80.56%, 76.19%, and 73.91% when the concentrations of NO_3_^−^ were 0, 0.01, 0.1, and 1 mmol L^−1^, respectively. Thus, it could preliminarily be determined that NO_3_^−^ might induce the generation of ·OH.

**Table 2:** Ketoprofen degradation kinetics, *k*_obs_, *k*_OH_, *R*_·OH_ and [·OH]

Items	[NO_3_^−^] (mM)	*K*_obs_ × 10^−2^ min^−1^		*k*_·OH_ (min^−1^)	*R*_·OH_ (%)	[·OH] (10^−11^mmol L^−1^)
		Without TBA	With TBA			
1	0	3.0 ± 0.126	0.3 ± 0.0111	2.7 ± 0.1149	90.00	5.10
2	0.01	3.6 ± 0.136	0.7 ± 0.0245	2.9 ± 0.1115	80.56	5.48
3	0.1	4.2 ± 0.105	1.0 ± 0.0435	3.2 ± 0.0615	76.19	6.05
4	1	4.6 ± 0.102	1.2 ± 0.0504	3.4 ± 0.0516	73.91	6.42

According to eqn (5), if the reaction rate constant between ketoprofen and ·OH was known, the steady-state concentration of ·OH in the system could be calculated. Therefore, a competitive experiment was conducted to obtain this constant. In this section, benzoic acid (BA) was selected as a probe in this competitive experiment to evaluate the reaction rate constant between ketoprofen and ·OH. The initial concentration of both ketoprofen and benzoic acid was 20 μmol L^−1^, and the pH value was adjusted to 10 to ensure a sufficient quantity of ·OH as well as to neglect the degradation caused by O_3_. The degradation rate constants of ketoprofen and benzoic acid were 2.9 × 10^−2^ and 1.9 × 10^−2^ min^−1^, respectively.

It is known that the reaction rate constant between benzoic acid and ·OH is 5.9 × 10^9^ M^−1^ S^−1^,^26^ according to eqn (5)–(7), and the reaction rate constant between ketoprofen and ·OH is calculated to be 8.82 × 10^9^ M^−1^ S^−1^, which coincided with results of previous research (8.4 × 10^9^ M^−1^ S^−1^).^15^ Therefore, the steady state concentration of ·OH could be calculated from eqn (8). As evidenced in Table 2, the concentration of NO_3_^−^ increased from 0 to 1 mmol L^−1^, and the steady state concentration of ·OH increased from 5.10 × 10^−11^ to 6.42 × 10^−11^ mmol L^−1^. These results affirmed that NO_3_^−^ could induce the generation of ·OH and that the concentration of ·OH increased with higher NO_3_^−^ concentrations within a certain range (0–1 mmol L^−1^).4
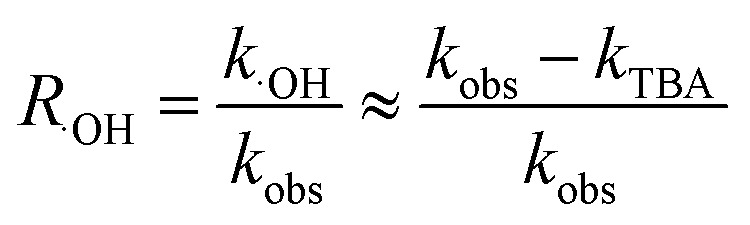
5
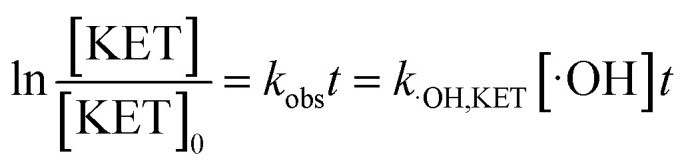
6
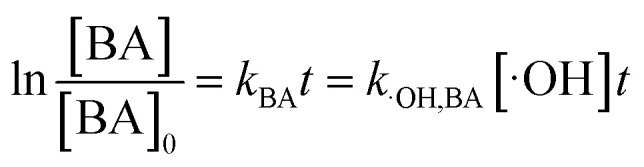
7
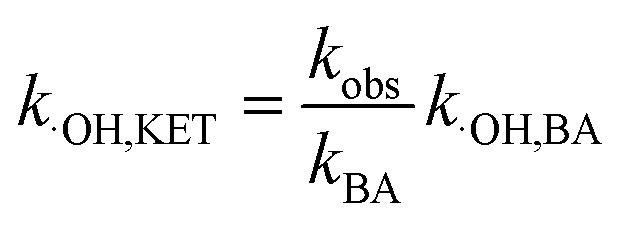
8
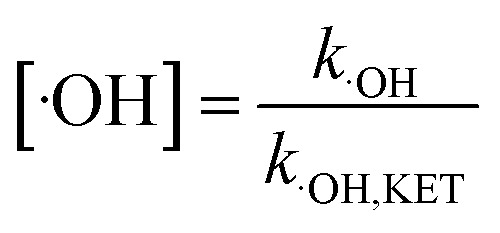
here, *R*_·OH_ is the contribution of ·OH, *k*_·OH_ is the degradation rate constant of ketoprofen with ·OH, *k*_TBA_ is the degradation rate of ketoprofen with the existence of TBA in the system, *k*_BA_ represents the overall degradation rate of benzoic acid in the system, [BA] is the molar concentration of benzoic acid at a specified time, *k*_˙OH,KET_ and *k*_·OH,BA_ are the reaction rate constants of ·OH with ketoprofen and benzoic acid, respectively, and [·OH] represents the steady state concentration of ·OH in the system.

### 3.2 By-products and degradation pathway speculation

This section summarizes the content mentioned above with the aim of further investigating the ketoprofen degradation mechanism under the presence of 1 mM nitrate ions as well as the identification of all degradation by-products.

**Fig. 3:**
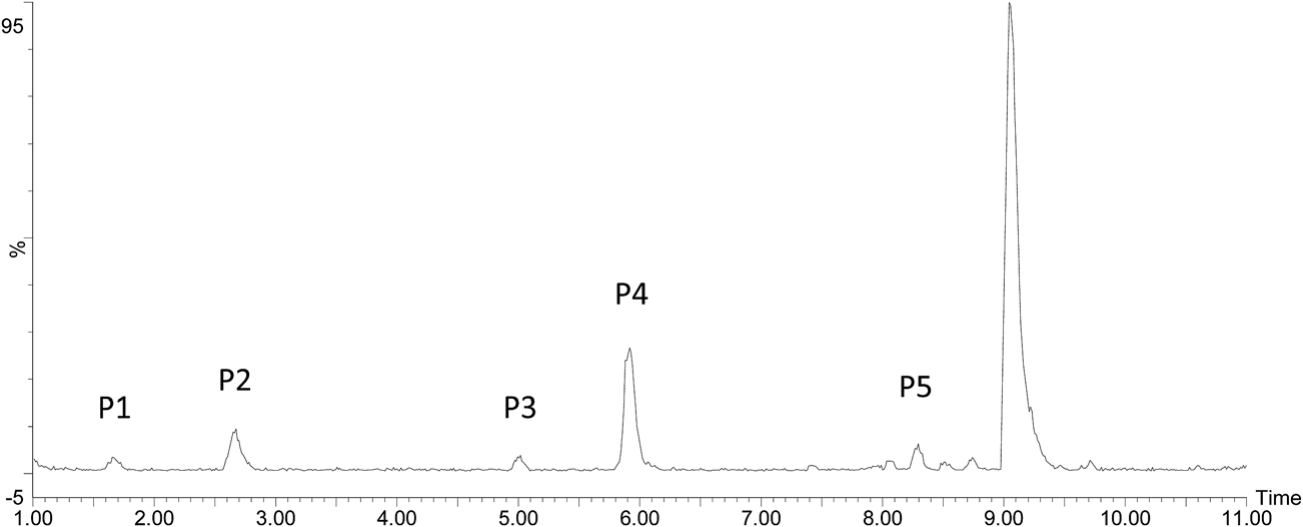
The total ion current of oxidation by-products.

**Fig. 4:**
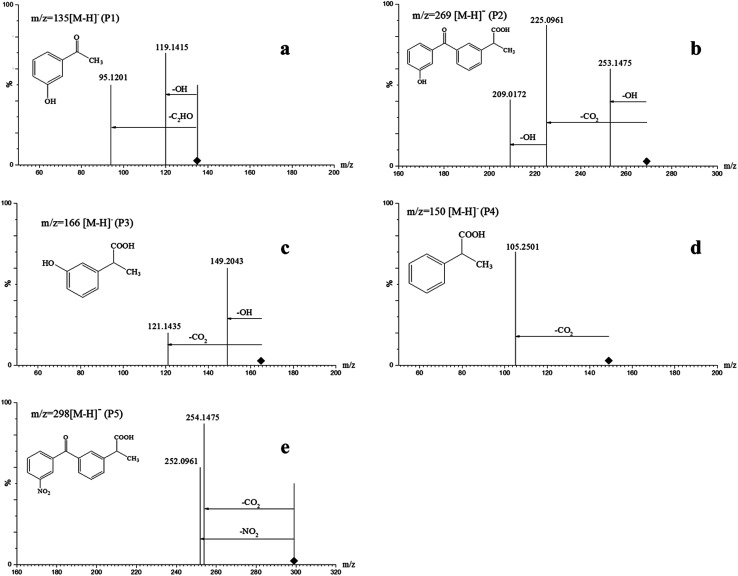
Fragment chart analyses of the secondary ion mass spectrometry of P1–P5.

The ketoprofen degradation products were analyzed by UPLC-Q-TOF-MS using the ESI negative ion mode, which allowed for the identification of a total of six compounds including the unaltered parent ketoprofen and five intermediates. The complete ion chromatograms are depicted in Fig. 3, with five major degradation by-product peaks at 1.68, 2.67, 5.00, 5.92, and 8.29 min assigned to P1, P2, P3, P4, and P5, respectively. Mass spectra and mass characteristics (*e.g.*, retention time, *m*/*z*, fragments, and prediction formula) of the above-mentioned substances are summarized in Table 3, whereas fragment chart analyses of the secondary ion mass spectrometry of the P1–P5 photolysis products are displayed in Fig. 4. Overall, the degradation of ketoprofen appeared to have at least four pathways: (1) hydroxylation on benzene ring pathway, (2) nitration on benzene ring pathway, (3) debenzophenone pathway, (4) ketonized pathway.

**Table 3:** Mass data obtained from the UPLC/MS/MS of ketoprofen oxidation by-products

Peak	Retention time (min)	ESI(−)MW	mDa	Fragments of MS^2^	Prediction formula
P1	1.68	136.1464 [M − H]^−^	−3.5	135, 119, 94	C_8_H_8_O_2_
P2	8.29	269.2845 [M − H]^−^	0.4	269, 254, 226, 210	C_16_H_14_O_4_
P3	5.00	165.1732 [M − H]^−^	2.8	165, 149, 121,	C_9_H_10_O_3_
P4	5.92	149.1746 [M − H]^−^	2.4	149, 105	C_9_H_10_O_2_
P5	7.41	298.2833 [M − H]^−^	−0.3	298, 252, 254	C_16_H_13_NO_5_

Due to the cogent electron-withdrawing effect of the carboxylic acid group and benzophenone group in the ketoprofen structure, the oxidation reaction preferentially occurred at the phenyl ring I rather than II. In addition, the meta-positions were easily activated and were vulnerable to attack, and this resulted in the formation of meta-by-products.^27^

**Fig. 5:**
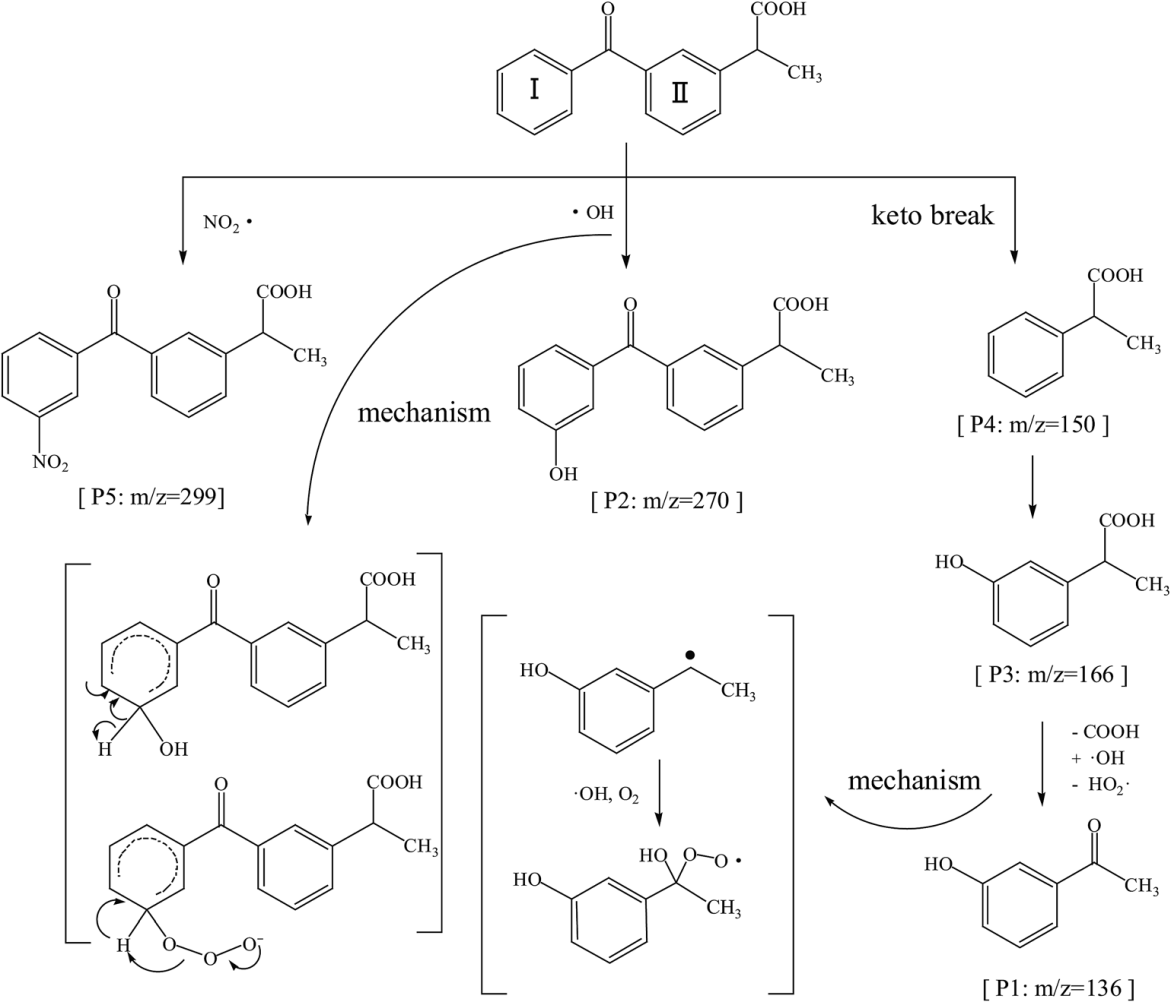
Possible oxidation pathways of KET in nitrate aqueous solution.

On one hand, the formula of P2 is C_16_H_14_O_4_, and the *m*/*z* was 270 with an additional O compared to those of the parents; major fragments at *m*/*z* 253(−16), 225(−44), and 209(−60) might correspond to the observed –OH loss, –COOH loss and the loss of both, respectively. These results resulted in the assumption that P6 was a monohydric derivative compound of ketoprofen degradation, which was formed from an electrophilic substitution (H by ·OH) on the aromatic ring. The mechanism of hydroxylation in ozonation is illustrated in Fig. 5 including the electrophilic substitution by O_3_ or nucleophilic attack by ·OH.^28^ Thus, Section 3.1 indicated that ·OH had a much higher contribution rate on the degradation of ketoprofen than O_3_ did. Thus, the nucleophilic substitution of ·OH in the benzene ring was inferred to be the dominant mechanism of benzene hydroxylation. As Illés^29^ mentioned in his study of the photo-induced degradation of ketoprofen in pure water, the hydroxylation by-products formed during the degradation of ketoprofen were preferentially due to the attack of ·OH on the benzene ring.

Furthermore, P5 (C_16_H_13_NO_5_, *m*/*z* 299) in the MS^2^ fragmentation spectrum of *m*/*z* 298 and the major fragments at *m*/*z* 252 (−46) and 254(−44) might correspond to the loss of –NO_2_ and –COOH. However, ·NO_2_ was generated according to eqn (2). The delocalization of a positive charge from ·NO_2_ to the aromatic ring brought about a strong electron-deficiency in the *meta*-positions on the aromatic ring,^30^ with a nitration-derivative of ketoprofen.

Apart from the two above-mentioned pathways, P4 (phenylpropionic acid, C_9_H_10_O_2_, *m*/*z* 149) was analyzed by UPLC-Q-TOF-MS at a retention time of 5.92 min, and it was defined as a by-product of ketoprofen debenzophenone pathway. Previous investigations were supportive of this pathway during the advanced oxidation of benzophenone-3.^31,32^ Subsequently, P3 compounds (*ortho*-hydroxybenzene propanoic acid, C_9_H_10_O_3_, *m*/*z* 166) were determined to be hydroxylated intermediates of P4 formed by undergoing further attacks on the meta-position of the benzene ring. Moreover, the –COOH group on the side-chain of P3 was observed to be further oxidized to form P1 (C_8_H_8_O_2_, *m*/*z* 136).

### 3.3 Toxicity measurement

**Fig. 6:**
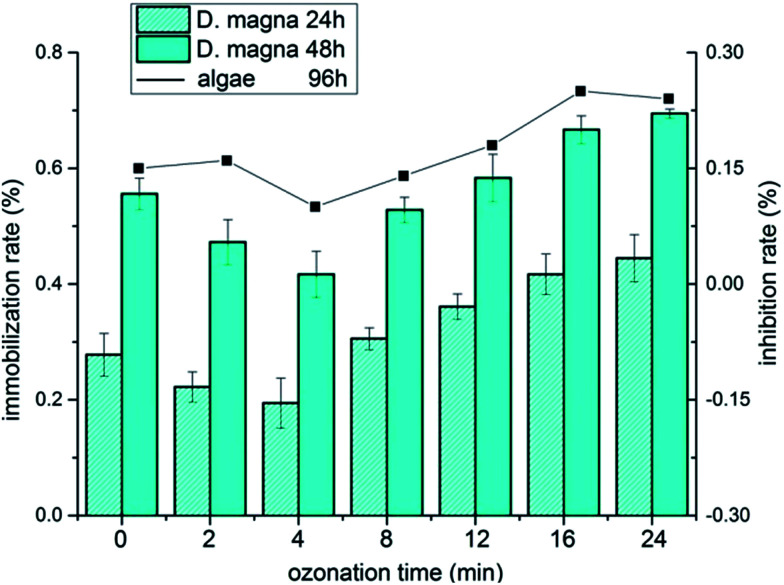
Variation of immobilization rates of *D. magna* in 24 h and 48 h (rectangle) and inhibition rate of algae in 96 h (line).

Toxicity measurement of organic pollutants is an essential indicator for practical wastewater treatment. As shown in Fig. 6, the initial immobilization rates of *D. magna* were 26.72% and 61.44% at 24 h and 48 h exposure, respectively. During the ozonation process, the immobilization rate was reduced slightly during treatment for 4 min. However, an evident increment occurred as the treatment proceeded and reached up to 50% and ultimately 80.56%. A similar trend was observed in the assay of algae, suggesting that the decomposition of initial KET led to the decrease of immobilization rate at the beginning. The accumulation of by-products resulted in the constant increase in the immobilization rate, indicating that the ecological risks of ozonation process are worthy of attention in practical treatment.

### 3.4 Ozonation of ketoprofen influenced by NO_2_^−^ and NH_4_^+^

**Fig. 7:**
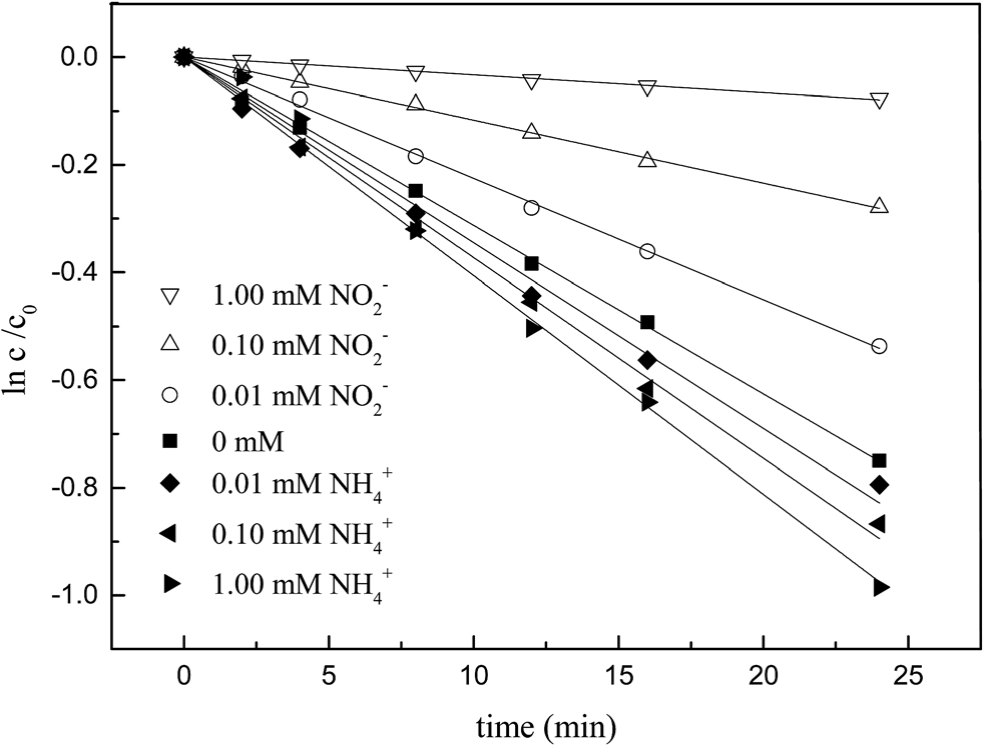
Effect of different concentrations of NO_2_^−^ and NH_4_^+^ on ozonation of KET.

It was revealed in Fig. 7 and Table 4 that NH_4_^+^ also had the capacity to accelerate the degradation of ketoprofen, which was similar to that of NO_3_^−^, but this capacity was less aggressive. On the contrary, NO_2_^−^ showed inhibitory effect in the degradation of ketoprofen, in that, as the NO_2_^−^ concentration increased, a stronger inhibition would occur. It was reported that the existence of NO_2_^−^ negatively affected the degradation of organic matter by AOPs.^18^

**Table 4:** Reaction rate of different concentration of NO_2_^−^ and NH_4_^+^

Species	NO_2_^−^				NH_4_^+^			
Concentration (mM)	0.00	0.01	0.10	1.00	0.00	0.01	0.10	1.00
Reaction rate (×10^−2^)	3.0	2.2	1.1	0.3	3.0	3.3	3.7	4.0

As for NH_4_^+^, previous studies have suggested that ozone might transform NH_4_^+^ to NO_3_^−^*via* the strong oxidizing capacity that directly converted N(−3) into N(+5) as revealed in eqn (9),^33–35^ thereby facilitating the degradation of organic matter in the same manner as that with NO_3_^−^.

9 NH_4_^+^ + 4O_3_ → NO_3_^−^ + 2H^+^ + H_2_O + 4O_2_

NO_2_^−^ comprises a type of reductive substance that has the capacity to perform redox reactions with substances in oxidation systems. This mechanism is elucidated in eqn (10) and (11), where the reaction rate constant between NO_2_^−^ and ozone molecules is 2.27 × 10^7^ M^−1^ S^−1^.^36^ According to eqn (12)–(14), the reaction rate constant between ketoprofen and O_3_ can be elucidated through competitive experiments using ibuprofen (IBP) as a probe. During the experiments, the initial concentrations of both ketoprofen and ibuprofen are 20 μmol L^−1^, and the degradation initiated by ·OH can be ignored *via* the addition of excess TBA and the adjustment of the pH value to 2. The degradation rate constant of ketoprofen is 0.5 × 10^−2^ min^−1^, whereas it is 3.3 × 10^−2^ min^−1^ for ibuprofen. It is known that the reaction rate constant between IBP and O_3_ is 7.2 M^−1^ S^−1^,^37^ and the reaction rate constant between ketoprofen and O_3_ is 1.09 M^−1^ S^−1^.

10 NO_2_^−^ + O_3_ → NO_3_^−^ + O_2_ 2.27 × 10^7^ M^−1^ S^−1^

11 NO_2_^−^ + ·OH → NO_2_· + OH^−^ (1–10) × 10^9^ M^−1^ S^−1^

12
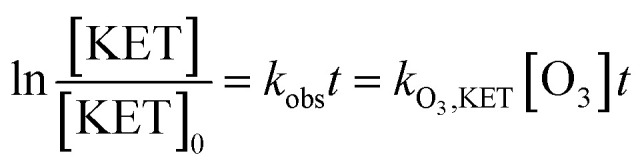
13
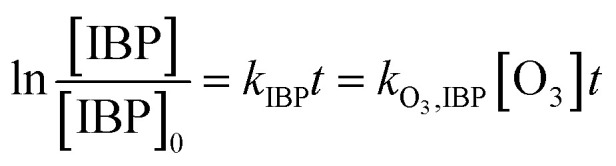
14
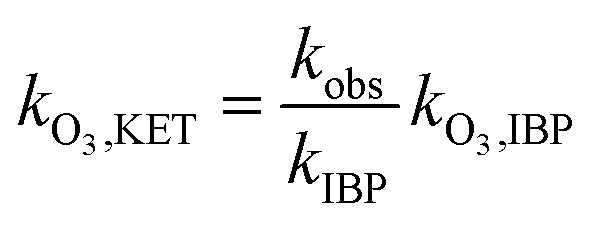
here, *k*_·O_3_,KET_ and *k*_·O_3_,BA_ are the reaction rate constants of O_3_ with ketoprofen and benzoic acid, respectively.

In comparison, the reaction rate of ozone molecules with NO_2_^−^ (2.27 × 10^7^ M^−1^ S^−1^) was much more rapid than that with ketoprofen (1.09 M^−1^ S^−1^). In this way, NO_2_^−^ restrained the ozonation of ketoprofen as the ozone molecule scavenger. On the other hand, both NO_2_^−^ and ketoprofen exhibited high reactive activities with ·OH, with reaction rate constants between (1–10) × 10^9^.^17,19,38^ The competition for ·OH between NO_2_^−^ and KET directly led to the decrease of the degradation rate of ketoprofen. As mentioned in Section 3.1, the contribution of ·OH to ketoprofen was approximately 90%, whereas the contribution of ·OH to ozone it was 10%. In this way, the competition for ·OH between NO_2_^−^ and KET was considered as the dominant factor in the reduction of the ketoprofen degradation rate.

## 4 Conclusions

The reaction kinetics and influence of different nitrogenous species on the ozonation of ketoprofen were investigated under simulated water disinfection conditions. Distinct discoveries demonstrated that the ketoprofen ozonation reactions aligned well with the pseudo first-order kinetics in the presence of NO_3_^−^. Five intermediates were simultaneously identified during the ozonation of ketoprofen, and four types of reaction pathways were speculated: hydroxylation and nitration on benzene ring, keto break, and ketonized reactions on the side chains. Furthermore, toxicity evaluation revealed that more harmful by-products were generated, which suggested that more attention should be paid to the wastewater disinfection process. Additionally, the experiment of NO_2_^−^ and NH_4_^+^ simply implied that the presence of nitrogenous species could influence the ozonation of ketoprofen.

## Conflicts of interest

There are no conflicts to declare.

## Supplementary Material
